# Exploring Epiploic Appendagitis: A Review

**DOI:** 10.7759/cureus.96996

**Published:** 2025-11-16

**Authors:** Katherine F Guijarro Falcon, Beebee Mubarak Jan, Hadeel Fathi Eltigani Suliman, Mohit Bhatia

**Affiliations:** 1 General Surgery, Princess Royal University Hospital, King's College Hospital London, Orpington, GBR

**Keywords:** acute abdomen, appendagitis epiploica, primary epiploic appendagitis (pea), treatment of appendagitis, unexplained abdominal pain

## Abstract

Epiploic appendagitis is a rare, self-limiting condition that typically presents with acute abdominal pain. Due to its uncommon nature, it is often overlooked and can sometimes be misdiagnosed. The condition has a nonspecific presentation, making clinical diagnosis challenging. However, it can be accurately diagnosed through radiological investigations. The presentation of epiploic appendagitis can vary, further complicating its clinical diagnosis. Fortunately, it is usually self-limiting and resolves with conservative management

## Introduction and background

Epiploic appendages are small, fat-filled outpouchings that project from the anti-mesenteric border of the entire colon, with the highest prevalence in the recto-sigmoid region [1]. Vesalius first described these appendages in 1543 [2]. These outpouchings have serosa coverings and are believed that these outpouchings remain small during childhood but enlarge with age progression [3]. It is hypothesized that they serve as a reservoir for blood in the colon. Some authors have suggested that they may play a role in mitigating local inflammation [4]. Although there is no definitive consensus on their physiological function, some studies propose that they act as a fat reservoir, potentially utilized during periods of low-calorie intake, while some consider their role in a defense mechanism against inflammation similar to the function of greater omentum [1,5].

Acute epiploic appendagitis (EA) is a benign, self-limiting inflammatory condition involving the appendages arising from the colonic walls that typically presents with variable and non-specific acute abdominal pain, ranging from mild to moderate or even severe, without many systemic features. EA is thought to occur due to torsion or inflammation of the epiploic appendage [6].

In 1956, Dockerty et al. were the first to describe EA as a clinical entity. Though rare, it is an important differential diagnosis as it can mimic other common conditions, presenting with acute abdominal discomfort that often leads patients to seek hospital care [7]. It is often an under recognized cause of acute abdominal pain. 

The incidence of epiploic appendagitis is higher in males, obese individuals, those who experience sudden weight loss, and those engaging in heavy physical exertion. It most commonly affects individuals in their fourth and fifth decades of life. Pain is typically localized, with the left lower quadrant being the most common site, though it can occasionally present on the right side. The pain is usually non-radiating, and patients are often able to pinpoint the site of the inflamed appendage [8].

Most patients present with localized pain and are otherwise systemically well. In some cases, a localized mass may be palpated, typically corresponding to the site of the inflamed segment of the appendage. In the majority of cases, blood tests are unremarkable, although mild leukocytosis and a slight elevation in C-reactive protein may be observed in certain instances, indicating early inflammation or localized ischemic fat necrosis [3].

Diagnosing this condition clinically can be challenging, as it often mimics other common conditions (Table 1) and typically presents with subacute or acute abdominal pain, bloating, a feeling of fullness, low-grade fever, and vomiting. There are no pathognomonic clinical features, making diagnosis difficult.

**Table 1:** Differential diagnosis of epiploic appendagitis Giannis et al. [5]

Condition
Appendicitis
Diverticulitis
Panniculitis
Ovarian torsion
Ovarian cyst rupture
Ectopic pregnancy
Pelvic inflammatory disease
Omental infarction
Fat containing tumours (e.g., angiomyolipoma)

**Figure 1:**
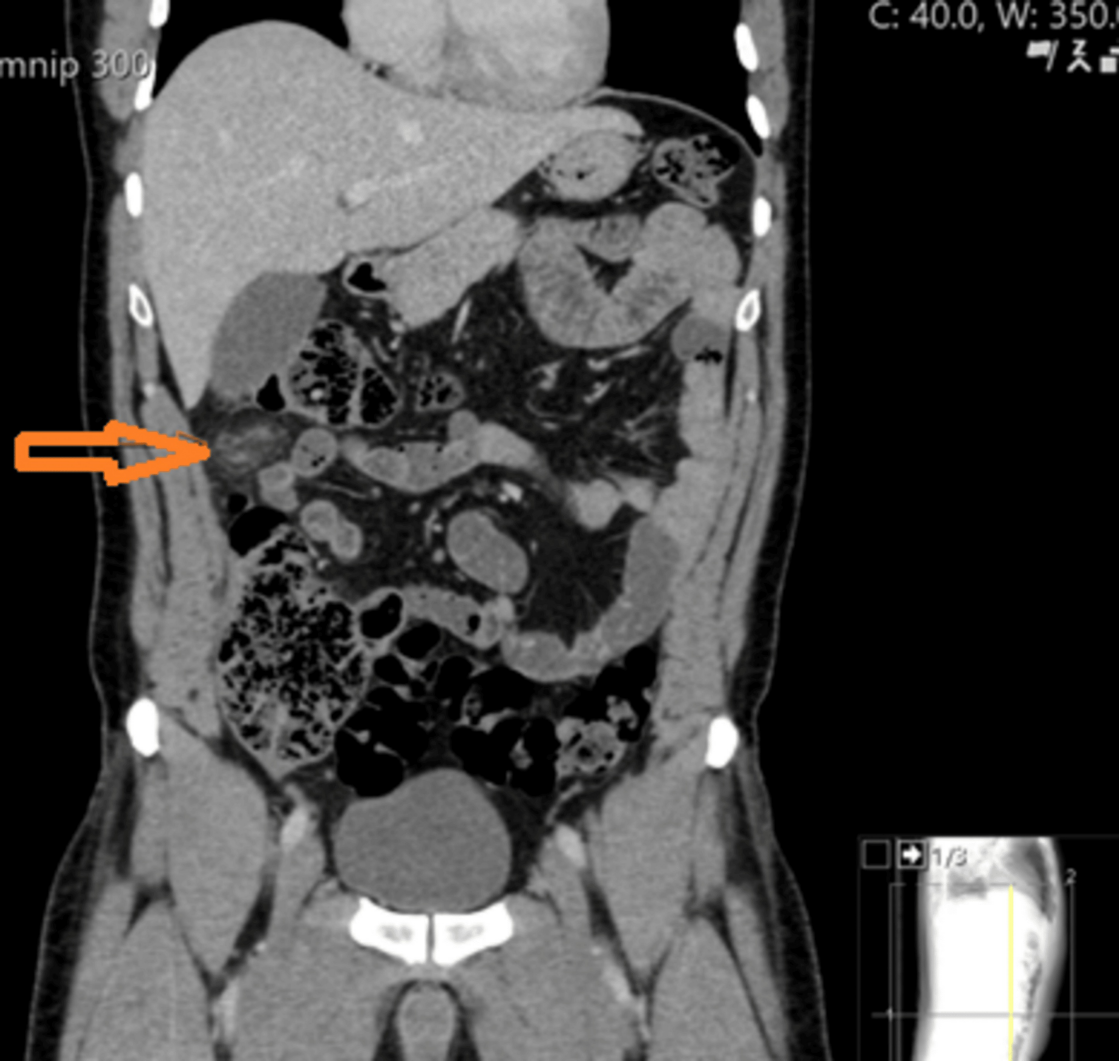
CT coronal view showing features of fat density ovoid structure with mild inflammatory fat stranding with some adjacent peritoneal thickening A 32-year-old male patient presented to our hospital with right upper abdominal pain (no patient identifiers on the CT film).

In most cases, it is discovered incidentally during a CT scan conducted to rule out other conditions. A CT scan is the most accurate diagnostic tool for identifying epiploic appendagitis, with a characteristic "central dot sign." [9] However, it can be difficult to distinguish a normal epiploic appendage on a CT scan due to its density overlapping with surrounding peritoneal and omental fat [10] (Figure 1).

## Review

Epidemiology and presentation

The exact incidence of epiploic appendagitis (EA) is unclear, but it has been reported in approximately 2-7% of patients presenting with suspected appendicitis or acute diverticulitis [11]. EA predominantly affects the sigmoid colon (about 50%) and is least commonly observed in the transverse colon. These appendages are present as a single row over the transverse colon and are nearly absent near the rectum [12].

These appendages receive blood through small arteries that originate from the vasa recta of the colon, while a small, tortuous vein drains them through a narrow pedicle. This setup makes them more prone to ischemia and torsion, which can result in infarction of these appendages, initiating the inflammatory process [1].

Historically, EA was diagnosed during surgical intervention. However, with the widespread use of CT scans in emergency and surgical departments, EA is now diagnosed in patients based on imaging characteristics. It is believed that these appendages are prone to ischemic changes due to limited vascular perfusion and enhanced mobility [13].

EA can be classified into primary and secondary types. Primary EA occurs due to torsion of the vascular pedicles, leading to ischemic infarction of these appendages. In contrast, secondary EA is diagnosed when the primary source of inflammation originates from other structures, such as appendicitis or diverticulitis. Other conditions, such as cholecystitis and inflammatory bowel disease (IBD), can also lead to secondary EA [14].

The clinical presentation of EA is nonspecific and often mimics other abdominal conditions. Most patients exhibit normal blood tests and vague clinical signs. Physical examination typically reveals mild to moderate abdominal pain, primarily in the lower left quadrant, although some cases may present with pain in the right lower quadrant. Occasionally, palpation may reveal a well-circumscribed mass with focal tenderness. In some cases, patients may show mildly elevated white blood cell counts and C-reactive protein levels due to the ongoing inflammatory process [15].

EA is often diagnosed incidentally while investigating other causes of abdominal pain. Most likely presumptive diagnosis includes appendicitis or diverticulitis, which are typically diagnosed with a dilated appendix and thickening of the colon or prominent pericolic fat stranding, respectively [16].

Diagnosis

Ultrasound Abdomen

Ultrasound abdomen (US) is a non-invasive, easily available investigation option free from radiation exposure, which can help in the diagnosis of EA. Typical findings include a non-compressible, solid ovoid lesion with surrounding inflammation denoted by a hypoechoic rim [3]. Kahveci et al. [17] highlighted the role of US in diagnosing EA. Its diagnostic value is limited by factors including the experience of the sonologist and the high body habitus of the patient, which may limit the confirmed diagnosis of EA.

CT Scans Abdomen

These appendages are not routinely seen on the CT scan due to indistinguishability from the surrounding adipose tissue; however, in the presence of inflammation, the resulting changes to the tissue density can help in identifying the inflamed appendages [18].

On CT, EA is seen as an oval-shaped lesion near the colonic wall with some surrounding inflammatory fat stranding and inflammation of the visceral peritoneum covering the appendage with a characteristic dot sign, which indicates a thrombosed vein. Usually, CT is sensitive in distinguishing EA from diverticulitis and omental infarction [19].

If a CT scan is inconclusive or contraindicated, an ultrasound (US) can be used to localize EA as a non-compressible oval mass, with no blood flow detected in the center on Doppler imaging [20].

MRI 

In pediatric and pregnant patients, MRI of the abdomen can be utilized to confirm the diagnosis. Though it is not routinely used due to limited access and high associated cost, it can be used in certain instances where CT is contraindicated, as it provides excellent resolution of the soft tissues. On MRI, EA appears as an ovoid mass with high signal intensity on both T1 and T2-weighted images, along with ring enhancement on T1-weighted images [20] (Table 2).

**Table 2:** Radiological findings of epiploic appendagitis Ng et al. [19]

CT Findings	Ultrasound Signs	MRI Findings
Fat density ovoid lesion (hyper-attenuating ring sign), mild bowel wall thickening, high-attenuation foci within fatty lesions	Non-compressible oval mass with lack of blood flow in the centre on Doppler; central dot-shaped hyperdense foci	Ovoid mass with high signal intensity in both T1- and T2-weighted images with ring enhancement on the T1 image

Differential Diagnosis

Patients with diverticulitis mostly have associated altered bowel habits, and in cases of complicated diverticular disease, they may present with associated urinary symptoms and be systemically unwell. Therefore, it is crucial to differentiate EA from complicated diverticular disease, as the latter can lead to severe complications like perforation and abscess formation, requiring more intensive treatment planning [21]. Diverticulitis can result in secondary EA; therefore, it is important to assess if both pathologies are present simultaneously.

Acute appendicitis is a frequent cause of acute abdominal pain and must be ruled out when diagnosing EA. It presents with distinctive signs and symptoms, including typical migratory pain. It is associated with prominent peritoneal irritation leading to rebound tenderness and elevated inflammatory markers, which aid in confirming the diagnosis [22].

Sometimes, distinguishing EA from omental infarction can be difficult due to similar features on imaging. However, omental infarction typically lacks the ring sign and is usually located farther from the colonic wall, which contrasts with the findings seen in EA. A study by Rao et al. retrospectively reviewed 660 CT scans and found that about 2% of patients had EA, with some cases being misdiagnosed as appendicitis or diverticulitis [23].

Treatment

Conservative Approach

The mainstay of treating EA remains symptomatic treatment, including analgesia. Certain studies have explored the use of antibiotics; however, there is no clear consensus on the routine use of antibiotics in treating EA owing to its self-limiting nature. It is believed that most of the patients have resolution of their symptoms in 2-3 weeks [14,15,24]. Therefore, attempts should be made to avoid any invasive interventions, and the focus should be on managing conservatively with symptom control. 

Surgical Approach

Usually, surgery is not indicated in managing EA; this approach is suitable in rare cases for patients developing recurrent symptoms or associated complications of bowel obstruction, intussusception, or abscess formation [5].

To date, very few reviews have been published on this topic due to its rare occurrence. A large study reported that around 26.4% patients underwent surgical intervention for EA, and around 5% patients developed wound infections. Surgical intervention involves ligation and excision of the affected appendage laparoscopically [25]. However, there is no consensus over the surgical approach, and it should only be considered in patients with a complicated course or recurrent symptoms. 

A study by Alhazmi et al. [27] on a review of 43 patients showed that EA was mostly confined to the left colon, and all the patients were managed with conservative treatment (inpatient and outpatient basis). Some of the few studies on EA have shown almost similar results and have been tabulated in the following table (Table 3).

**Table 3:** Selected review articles describing EA Alhazmi et al. [27], Ortega-Cruz et al. [28], Thomas et al. [29], van Breda Vriesman et al. [30], Vasquez et al. [31], Tan et al. [32], Nugent et al. [33] EA: epiploic appendagitis

Authors	N (sample size)	Time period included	Outcome/Results
Ortega-Cruz et al. [28]	8	2007-2009	Mean age, 58 years; risk factors, male, overweight; 75% patients presented with left iliac fossa pain; majority were managed with conservative management.
Alhazmi et al. [27]	43	2010-2022	Males were affected more than women; 76.7% patients had CT as a diagnostic for EA; all patients were treated with analgesia (non-steroidal anti-inflammatory drugs)
Thomas et al. [29]	208	1974	73% cases developed EA due to torsion or inflammation of the appendage.
van Breda Vriesman et al. [30]	49	2003	69-89% patients had left lower quadrant pain as presenting symptoms; only 2.5% patients were correctly diagnosed clinically/pre-operatively.
Vázquez et al. [31]	73	2007-2013	Mean age 45 years; 60% patients had ultrasonography for diagnosis; 92% patients were treated with NSAIDS; 2 patients underwent laparoscopy.
Tan et al. [32]	78	2009-2019	Male predominance (55%); most common associated co-morbidity, diabetes mellitus (n=10); sigmoid, most common affected site (41%); opioids reduced re-hospitalisation; 6% patients diagnosed intra-operatively.
Nugent et al. [33]	100	Unknown	Mean age 50.12 years; 67 males affected; sigmoid colon most affected, association with increased abdominal adipose tissue.

In rare cases where EA is diagnosed during surgery or laparoscopy, it can be managed by excising the affected segment and performing sero-muscular inversion of the site. In more complicated cases, where abscess formation or intussusception occurs, surgical intervention becomes the primary treatment [33].

It is important to highlight the value of thorough clinical history and examination with an aim to narrow down the differentials and use the appropriate investigations to enhance patient care and safety. This is all the more applicable when dealing with less frequent causes of abdominal pain, which can be challenging to identify and treat.

## Conclusions

This is a rare, self-limiting condition that can be challenging to diagnose. It typically presents as sudden, localized pain and, in some cases, may manifest as a palpable localized mass. Laboratory investigations usually return normal results. Clinicians should consider this condition as a differential diagnosis, particularly when examination findings and blood tests are unremarkable. Imaging plays a crucial role in diagnosis, but it is important to rule out other potentially serious causes of acute abdominal pain that may require surgical intervention. Recognizing this condition early can prevent unnecessary hospital admissions and treatments, as most cases complete with conservative management. CT scan is sensitive and quite informative in its diagnosis. However, in pregnant patients and children, MRI and US can be beneficial. Early recognition, along with patient awareness, can improve care and provide reassurance. Resolution of clinical symptoms with conservative and symptomatic management typically obviates the need for formal follow-up; nevertheless, careful safety-netting is crucial to promptly identify any potential progression of symptoms. Awareness of this rare but significant condition is vital to reduce healthcare burdens, lower associated costs, and minimize the risk of medical litigation for healthcare professionals. Initiatives should be taken by clinicians to discuss less encountered clinical cases in their departmental meetings, which will help in enhancing clinical acumen and decision-making of the budding medical professionals. 
